# ASPIC: a novel method to predict the exon-intron structure of a gene that is optimally compatible to a set of transcript sequences

**DOI:** 10.1186/1471-2105-6-244

**Published:** 2005-10-05

**Authors:** Paola Bonizzoni, Raffaella Rizzi, Graziano Pesole

**Affiliations:** 1 DISCo, University of Milan Bicocca, via Bicocca degli Arcimboldi, 8, Milan, 20135, Italy.; 2 Dipartimento di Scienze Biomolecolari e Biotecnologie, University of Milan, via Celoria, 26, Milan, 20133, Italy.

## Abstract

**Background:**

Currently available methods to predict splice sites are mainly based on the independent and progressive alignment of transcript data (mostly ESTs) to the genomic sequence. Apart from often being computationally expensive, this approach is vulnerable to several problems – hence the need to develop novel strategies.

**Results:**

We propose a method, based on a novel multiple genome-EST alignment algorithm, for the detection of splice sites. To avoid limitations of splice sites prediction (mainly, over-predictions) due to independent single EST alignments to the genomic sequence our approach performs a multiple alignment of transcript data to the genomic sequence based on the combined analysis of all available data. We recast the problem of predicting constitutive and alternative splicing as an optimization problem, where the optimal multiple transcript alignment minimizes the number of exons and hence of splice site observations.

We have implemented a splice site predictor based on this algorithm in the software tool ASPIC (Alternative Splicing PredICtion). It is distinguished from other methods based on BLAST-like tools by the incorporation of entirely new ad hoc procedures for accurate and computationally efficient transcript alignment and adopts dynamic programming for the refinement of intron boundaries. ASPIC also provides the minimal set of non-mergeable transcript isoforms compatible with the detected splicing events. The ASPIC web resource is dynamically interconnected with the Ensembl and Unigene databases and also implements an upload facility.

**Conclusion:**

Extensive bench marking shows that ASPIC outperforms other existing methods in the detection of novel splicing isoforms and in the minimization of over-predictions. ASPIC also requires a lower computation time for processing a single gene and an EST cluster. The ASPIC web resource is available at .

## Background

The completion of several genome projects has, rather surprisingly, revealed that despite a remarkable heterogeneity in organism complexity and genome size, the variation in total gene number is much less pronounced, with a less than a 10-fold increase in gene number between prokaryotes (e.g. *E. coli*) and vertebrates (e.g. human) [1].

However, the level of protein complexity in humans and other vertebrates is much higher than expected from the estimated gene number. Alternative splicing, leading to the generation of multiple transcripts from single genes, is believed to be the major mechanism expanding protein diversity in higher organisms [2]. These transcripts can differ both in the untranslated (UTR) and in coding regions. Thus, using a different combination of donor and acceptor splice sites, transcripts encoding different proteins can be produced with alternative UTRs regulating their fate in the cell. Indeed, recent large scale genomic studies have shown that alternative splicing occurs in 40–60% of human genes [3] and that it is a likely determinant of species-specificity since an unexpectedly low level of alternative splicing pattern conservation has been observed in pairs of orthologous genes [4]. Recent studies have also shown that alternative splicing is important for determining developmental- and tissue-specific- gene expression [5,6]. Aberrant splicing forms are also associated with human diseases [7]. For these reasons, there is a growing interest in the high-throughput identification of alternative splicing forms in human and other organisms [8].

Recently, there has been a growing interest in the design of computational methods to predict alternative splicing. Published methods may be classified in three groups: methods based on the comparison of expressed sequences to each other (i.e. [9], [10], [11]), methods based on the alignment of ESTs to the genomic sequence [12-14] and more recently methods that combine the previous two approaches, i.e. EST comparison and genome comparison, as proposed in [15] and [16]: we call such methods *multiple EST alignment methods*. A wide ranging discussion of the limitations of the first two methods has been presented and it has been shown that combining the two approaches leads to clear improvements in alternative splicing identification [16]. Computational methods may be also classified according to the computational approach used to produce EST alignments. Indeed, it must be pointed out that the majority of tools uses BLAST, sim4 or most recently BLAT to map ESTs to the genome (see Table 1 in [11]). These tools are often error prone when aligning ESTs because they have not been designed to consider either the relationship between ESTs and their corresponding genomic sequences or sequencing errors in ESTs – for example the presence of large gaps, short exons or specific constraints on the alignment near intron boundaries.

**Table 1:** Benchmark comparison of ASPIC with other similar tools

	ASPIC			ASAP		ASD		ACEVIEW	
				
*GENE*	*#introns (#novel)*	*#TS*	*#EST/splice*	*#introns (#ASPIC)*	*#TS*	*#introns (#ASPIC)*	*#TS*	*#introns (#ASPIC)*	*#TS*
ABCB10	12(0)	2	12.42	12(12)	1	Not Found		13(12)	3
ACADM	21(1)	15	31.52	15(14)	6	Not Found		22(20)	14
ACTN2	23(0)	28	19.09	20(20)	1	22(22)	4	23(23)	8
ADAM15	41(4)	67	40.07	13(13)	4	29(29)	11	56(37)	25
ADAMTS4	8(0)	3	8.63	7(7)	1	8(8)	4	8(8)	3
ADORA1	13(1)	10	4.69	8(8)	4	12(12)	9	10(9)	7
ADORA3	15(13)	5	6.13	2(2)	2	Not Found		3(2)	3
AGL	40(1)	12	14.48	38(38)	1	Not Found		39(39)	10
AGRN	41(4)	21	16.98	35(32)	1	Not Found		45(37)	11
AGT	7(2)	17	52.86	4(3)	1	Not Found		9(5)	8
AHCYL1	26(4)	35	48.50	19(19)	5	19(19)	4	26(22)	13
AKR7A2	9(1)	6	73.33	6(6)	1	7(7)	2	31(8)	15
ALDH9A1	17(4)	10	39.29	11(11)	2	Not Found		16(13)	7
ALPL	15(0)	8	19.47	14(13)	3	13(13)	3	17(15)	9
AMPD1	14(0)	4	7.64	13(13)	1	Not Found		45(14)	12
ANGPTL1	6(0)	3	10.50	5(5)	1	Not Found		6(6)	4
ANGPTL3	6(0)	5	19.17	6(6)	2	Not Found		8(6)	7
ANXA9	15(1)	3	14.40	13(13)	1	14(13)	2	16(14)	6
AP4B1	18(0)	22	14.61	12(12)	1	17(16)	12	16(16)	14
APCS	2(1)	5	62.50	1(1)	1	Not Found		1(1)	1
ARHGEF2	32(1)	37	15.19	22(22)	3	26(25)	6	35(31)	17
ARHGEF11	47(1)	9	7.70	42(42)	2	41(40)	6	46(45)	17
ARHGEF16	14(2)	12	18.64	10(10)	1	Not Found		15(12)	5
ARNT	26(1)	14	11.73	20(18)	1	22(21)	3	38(26)	14
ARPC5	4(1)	2	120.75	3(3)	1	4(2)	2	6(3)	4
ARTN	8(0)	10	5.25	7(7)	4	6(6)	3	7(7)	10
ATAD3A	22(2)	10	36.41	16(16)	2	Not Found		58(21)	27
ATP1B1	11(2)	12	59.27	7(7)	1	10(9)	4	11(8)	10
ATP2B4	29(3)	11	8.07	22(22)	5	23(23)	3	26(26)	14
Clorf10	2(0)	1	7.00	2(2)	1	Not Found		2(2)	2
Clorf26	22(0)	7	7.91	17(17)	1	Not Found		23(22)	6
C1QB	3(1)	3	25.67	5(2)	3	5(2)	3	6(2)	5
CAPZA1	15(4)	15	68.40	11(11)	2	10(9)	3	12(11)	7
CTRC	9(1)	4	36.22	8(8)	1	Not Found		8(7)	3
DMRTA2	2(0)	2	1.00	1(1)	1	Not Found		2(2)	1
DPH2L2	12(1)	11	31.25	10(10)	7	12(11)	12	12(11)	14
EPHA2	20(1)	8	13.45	16(16)	1	17(17)	7	20(19)	8
EYA3	20(0)	9	11.40	15(15)	1	Not Found		21(20)	10
FBXO2	9(0)	6	13.67	9(8)	2	6(5)	3	9(8)	5
FCGR3B	7(0)	5	22.57	4(4)	1	Not Found		7(7)	6
FUCA1	11(3)	8	18.00	8(8)	2	Not Found		7(7)	2
GBP2	17(3)	8	27.82	12(12)	2	Not Found		26(14)	10
GMEB1	12(1)	5	16.67	9(9)	2	11(11)	3	11(11)	6
HNRPR	20(2)	38	45.70	16(15)	7	12(12)	7	21(18)	17
LGALS8	22(2)	25	16.00	12(11)	4	13(13)	4	27(19)	21
LRRN5	3(0)	3	3.00	5(3)	2	Not Found		5(3)	4
LYPLA2	15(0)	14	96.07	14(14)	6	Not Found		15(14)	14
MASP2	11(0)	5	7.00	11(11)	2	11(11)	5	11(11)	6
MOV10	35(4)	42	25.29	29(29)	7	24(23)	8	33(31)	21
NPPB	2(0)	1	30.50	2(2)	1	Not Found		3(2)	3
PAFAH2	18(3)	11	15.06	12(11)	2	13(13)	5	16(14)	8
PALMD	8(0)	9	35.63	7(7)	1	Not Found		9(8)	8
PEX10	12(1)	13	21.58	6(6)	1	10(10)	7	12(10)	11
PINK1	10(2)	15	40.20	7(7)	2	Not Found		9(8)	10
PTPRU	38(3)	15	12.89	20(20)	1	Not Found		35(35)	9
RHOC	17(3)	5	8.35	13(2)	7	15(1)	9	39(14)	31
SDC3	6(2)	5	5.33	4(3)	2	8(4)	5	9(5)	6
SDHB	11(0)	12	97.27	9(9)	3	Not Found		13(11)	11
SERPINC1	12(2)	7	18.75	8(8)	2	8(8)	3	16(10)	11
SFPQ	12(3)	11	74.75	9(9)	1	9(9)	3	17(9)	25
TARDBP	21(3)	20	29.38	15(13)	4	9(9)	4	18(16)	15
TCN2	13(1)	10	26.15	9(9)	2	12(12)	4	13(10)	11
TOR3A	15(2)	9	19.20	9(9)	4	11(11)	8	15(13)	12
VAMP3	5(0)	3	80.60	6(5)	2	6(5)	3	7(5)	10
Total	1009(94)	11.9	28.3	753(721)	2.3	495(461)	5.1	1194(905)	9.7

ASPIC results from a random sample of 64 human genes from Chromosome 1 compared to those from the ASAP, ASD and AceView resources. The first column reports the HUGO name of the examined gene. The ASPIC data include the total number of predicted introns (novel introns in brackets), the minimum number of compatible transcripts and the average number of ESTs supporting gene splices. Introns with 2 non canonical splices are accepted by ASPIC only if confirmed by at least two ESTs. For the other resources the number of predicted introns (in brackets those also predicted by ASPIC) and the minimum number of compatible transcripts are reported. Other resources: AceView (July 2003 and August 2004 releases), ASD (July 2004) and ASAP (July 2004).

In this paper we propose a method that is not based on traditional BLAST-like (or BLAT-like as in [17]) alignment tools for spliced alignment, but which relies on a new heuristic for multiple EST alignments that allows – as in [12] – the use of a high number of insertions/deletions and specific scoring criteria for the spliced alignment in order to generate more accurate splice site predictions (see [18]). Indeed, even recent tools such as BLAT [19] produce erroneous alignments when used for EST-genome comparison as observed in [17] and require further corrections to the alignments produced. For example BLAT tends to create many small gaps in the alignment in cases of low sequence quality.

Through a combined analysis of all EST data and their genomic alignments our heuristic method aims to reduce over predictions of splice sites due to EST sequence errors or erroneous single EST alignments. This goal is achieved by minimizing the set of splice sites that is compatible with a multiple alignments of all transcript data. This approach overcomes the limitations of methods that (incorrectly) assume independency of single transcript-genome alignments. Indeed, tools based on independent single EST alignments (for example, Spidey [14] and Squall [20]) may produce false splice forms that would not be supported by a combined multiple alignment of all ESTs against the genomic sequence.

## Implementation

### Methods

Our method is based on the formalization of the problem of detecting splice sites as an optimization problem (Multiple EST Factorization Compatibility, MEFC) as proposed in [15]: it implements an heuristic that extends – and greatly improves – a basic algorithmic approach proposed by the same authors in [15]. An evident shortcoming of computational methods to predict splice sites is represented by the large number of false positive predictions produced by these methods. To overcome this limitation, we propose that an optimization criterion may be required to construct a multiple transcript alignment: the objective function of such a criterion is to minimize the number of exon predictions and hence of alignment-inferred splice sites. There is theoretical evidence for this assumption which is also supported by several real cases encountered while analyzing EST alignments. Indeed, such an optimization criterion is required when there are multiple possible adequate alignments of an EST region (or candidate exon) to the genomic sequence, even when restrictive rules are used (i.e. *GT *– *AG *splice sites) to restrict the alignment to biologically plausible solutions. The use of the optimization criterion, the combined EST analysis and the fact that our method is entirely based on a novel alignment procedure all differentiate our approach from those previously presented. The method we propose here is also different from the ones suggested in [21] and [11] where a combined analysis of EST alignments is done after all EST alignments have been generated. The method we propose also aims to reduce the computational time as in [20], while retaining a high accuracy of predictions. It is specifically designed to process a whole gene and large number of ESTs – the databases currently contain about 6 millions human ESTs and the number is growing rapidly. As shown in [20], computational times for a single EST alignment may range from a fraction of a second to the several seconds required by programs such as sim4 [22].

The software tool ASPIC (Alternative Splicing PredICtion) has been designed and implemented in a user-friendly web-server accepting as input a gene sequence and transcript data, typically a Unigene cluster related to the gene. Major features of ASPIC include its applicability to the analysis of splice variants in several organisms, and the fact that it collects together several sources of information on splice sites in a single web-based tool.

ASPIC also provides a minimal set of transcript isoforms explaining all alternative splice events occurring among the set of transcripts considered. Furthermore, it includes a module for detecting and scoring splice junctions (canonical and non-canonical) by using quality measures based on [18] and [23]. An extensive benchmark comparison of ASPIC with respect to other similar tools [24,25] shows that our method calculates the location of splice sites with high sensitivity and accuracy but still retaining an high computational efficiency such that in [20]. Remarkably, ASPIC differently from [20] combines EST alignment to splice site prediction.

### Algorithm overview

In the following, we will use the term *EST *to denote a transcript and *genomic sequence *to refer to a gene related to a set of transcripts. We will use *G *to denote a genomic sequence, that is, a sequence over alphabet Σ = {A, C, G, T} ∪ {N}, with N denoting any nucleotide. Genomic sequences containing sequence repeats or short exons may be alignable to the same EST sequence in a number of equally probable ways. This fact further complicates the problem of identifying the correct exon-intron structure. However, it is reasonable to assume that a correct exon-intron structure can be obtained by aligning all EST sequences so that regions that are common to different ESTs are aligned to the same region of the gene. This assumption leads to the framing of the problem of predicting gene structure from a set of ESTs as an optimization problem as introduced in [15] with the MEFC problem (Minimum EST Factorization Compatible with a genomic sequence). In this context, the gene structure prediction problem has an instance consisting of a set of EST sequences and a genomic sequence: the question is to compute the constitutive exons of the genomic sequence and the factorization of each EST into such genomic exons with the objective of minimizing the number of predicted exons.

In fact, as illustrated in the examples below, a minimum length exon-factorization of a genomic sequence would forbid multiple unsupported EST alignments. However, with real data, situations frequently occur where multiple EST alignments are generated and additional criteria to find an exon-factorization are required, thus justifying (as discussed in the following sections) the use of the optimization criterion in our method.

**Figure 1:**
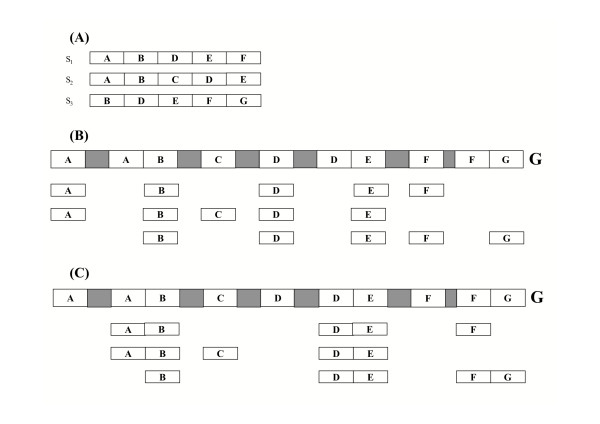
The figure illustrates two gene-factorizations into 7 and 4 pseudo-exons of the genomic sequence *G*. Let *S*_1_, *S*_2 _and *S*_3 _be EST sequences in S agreeing to the genomic sequence *G*, where sequence *S*_1 _= *ABDEF*, *S*_2 _= *ABCDE *and *S*_3 _= *BDEFG*, each letter in {*A*, *B*, *C*, *D*, *E*, *F*, *G*} denotes a sequence (A). In (B) and (C) two alternative EST-genome alignments of sequences *S*_1_, *S*_2 _and *S*_3 _are represented: each EST factorization of *S*_*i *_associated with the EST-genome alignment is shadowed. Pseudo-exons in the gene-factorization are colored white, while introns are in grey. Segments labelled by letters represent regions of the genomic sequence that align to a substring of the input sequence of the corresponding letter. Note that an approach that aligns independently each sequence *S*_1_, *S*_2 _and *S*_3 _to *G*, one after the other, may produce the gene-factorization <*A*, *B*, *C*, *D*, *F*, *E*, *G*> consisting of 7 pseudo-exons (B), while the one minimizing the number of pseudo-exons provides only 4 pseudo-exons (C). Indeed, there are EST factorizations of each *S*_*i *_that are compatible or variant compatible with the gene-factorization *G*_*E *_= <*AB*, *C*, *DE*, *FG*>. More precisely, <*AB*, *DE*, *F*> is an EST-factorization of *S*_1 _that is compatible to *G*_*E*_. Then <*AB*, *C*, *DE*> is an EST-factorization of *S*_2 _compatible to *G*_*E*_. Finally, <*B*, *DE*, *FG*> is an EST-factorization of *S*_3 _compatible with *G*_*E *_(C).

1. Terminal EST factors may be short (10–30 bp in length) and may have multiple plausible alignments to the genomic sequence, particularly when the EST sequence contains errors.

2. Part of a factor may be repeated along the genomic sequence. A theoretical example of this situation, and how optimization may be used to find correct predictions, is reported in Fig 1. Additional file 1 illustrates a specific example of this situation, occurring in the Unigene cluster related to the human AMY2A gene.

3. Short repeats may occur in the genomic sequence and EST sequences may contain errors near splice junctions.

### The MEFC problem: definition

In the following we introduce some basic notions that allow us to define the MEFC problem and describe the method we propose to face it.

We recall that there are four main patterns of alternative splicing that potentially may occur in nature [2]:

1) exon-skipping; 2) mutually exclusive exons; 3) competing 5'/3' ends; and 4) intron retention. While the first two splicing modes simply determine whether an exon is used or not during splicing, in the third mode the transcript *splicing variants *derive from competing partially overlapping exons. Finally, intron retention occurs when an exon is present in a transcript, while in another it appears with a missing internal region.

Then, a *gene factorization G*_*E *_of *G *is a sequence <*f*_1_, ..., *f*_*n*_> of *n *substrings *f*_*i *_of *G*, we define *pseudo-exons*, such that *G *is given by the concatenation of the pseudo-exons *f*_*i *_interspersed by other substrings called *introns*. In particular, a pseudo-exon defines a contiguous genome region corresponding to and/or containing one or more exon splice variants.

An *EST factorization *of an EST sequence *S *is an ordered sequence <*s*_1_, *s*_2_, ..., *s*_*k*_> such that *S *= *s*_1_*s*_2 _... *s*_*k*_, where each substring *s*_*i *_is called a *factor *of the EST *S*. The *edit distance ed*(*x*, *y*) between two sequences *x *and *y *measures the number of mismatches in the alignment of *x *and *y*.

We define an EST factorization <*s*_1_, *s*_2_, ..., *s*_*k*_> *compatible with *a gene-factorization *G*_*E *_of a genomic sequence *G *if there exists a sequence of genomic pseudo-exons fi1,fi2,fi3,…,fik
 MathType@MTEF@5@5@+=feaafeart1ev1aaatCvAUfKttLearuWrP9MDH5MBPbIqV92AaeXatLxBI9gBaebbnrfifHhDYfgasaacH8akY=wiFfYdH8Gipec8Eeeu0xXdbba9frFj0=OqFfea0dXdd9vqai=hGuQ8kuc9pgc9s8qqaq=dirpe0xb9q8qiLsFr0=vr0=vr0dc8meaabaqaciaacaGaaeqabaqabeGadaaakeaacqWGMbGzdaWgaaWcbaGaemyAaK2aaSbaaWqaaiabigdaXaqabaaaleqaaOGaeiilaWIaemOzay2aaSbaaSqaaiabdMgaPnaaBaaameaacqaIYaGmaeqaaaWcbeaakiabcYcaSiabdAgaMnaaBaaaleaacqWGPbqAdaWgaaadbaGaeG4mamdabeaaaSqabaGccqGGSaalcqWIMaYscqGGSaalcqWGMbGzdaWgaaWcbaGaemyAaK2aaSbaaWqaaiabdUgaRbqabaaaleqaaaaa@41F0@ of *G *such that for each factor *s*_*j*_, with 2 ≤ *j *≤ *k *- 1, *ed*(*s*_*j*_, fij
 MathType@MTEF@5@5@+=feaafiart1ev1aaatCvAUfKttLearuWrP9MDH5MBPbIqV92AaeXatLxBI9gBaebbnrfifHhDYfgasaacH8akY=wiFfYdH8Gipec8Eeeu0xXdbba9frFj0=OqFfea0dXdd9vqai=hGuQ8kuc9pgc9s8qqaq=dirpe0xb9q8qiLsFr0=vr0=vr0dc8meaabaqaciaacaGaaeqabaqabeGadaaakeaacqWGMbGzdaWgaaWcbaGaemyAaK2aaSbaaWqaaiabdQgaQbqabaaaleqaaaaa@311D@ ) is bounded by a given parameter *bound*, factors *s*_1 _and *s*_*k *_differ from a suffix of pseudo-exon fi1
 MathType@MTEF@5@5@+=feaafiart1ev1aaatCvAUfKttLearuWrP9MDH5MBPbIqV92AaeXatLxBI9gBaebbnrfifHhDYfgasaacH8akY=wiFfYdH8Gipec8Eeeu0xXdbba9frFj0=OqFfea0dXdd9vqai=hGuQ8kuc9pgc9s8qqaq=dirpe0xb9q8qiLsFr0=vr0=vr0dc8meaabaqaciaacaGaaeqabaqabeGadaaakeaacqWGMbGzdaWgaaWcbaGaemyAaK2aaSbaaWqaaiabigdaXaqabaaaleqaaaaa@30B0@ and a prefix of fik
 MathType@MTEF@5@5@+=feaafiart1ev1aaatCvAUfKttLearuWrP9MDH5MBPbIqV92AaeXatLxBI9gBaebbnrfifHhDYfgasaacH8akY=wiFfYdH8Gipec8Eeeu0xXdbba9frFj0=OqFfea0dXdd9vqai=hGuQ8kuc9pgc9s8qqaq=dirpe0xb9q8qiLsFr0=vr0=vr0dc8meaabaqaciaacaGaaeqabaqabeGadaaakeaacqWGMbGzdaWgaaWcbaGaemyAaK2aaSbaaWqaaiabdUgaRbqabaaaleqaaaaa@311F@ , respectively, by a number of alignment mismatches bounded by *bound*.

Because of alternative splicing, we further provide the notion of EST factorization *variant compatible *with a gene-factorization *G*_*E*_. This is simply obtained by requiring in the previous notion that *ed*(*s*_*j*_, *factor*(fij
 MathType@MTEF@5@5@+=feaafiart1ev1aaatCvAUfKttLearuWrP9MDH5MBPbIqV92AaeXatLxBI9gBaebbnrfifHhDYfgasaacH8akY=wiFfYdH8Gipec8Eeeu0xXdbba9frFj0=OqFfea0dXdd9vqai=hGuQ8kuc9pgc9s8qqaq=dirpe0xb9q8qiLsFr0=vr0=vr0dc8meaabaqaciaacaGaaeqabaqabeGadaaakeaacqWGMbGzdaWgaaWcbaGaemyAaK2aaSbaaWqaaiabdQgaQbqabaaaleqaaaaa@311D@ )) is bounded by a given parameter *bound*, where *factor *(fij
 MathType@MTEF@5@5@+=feaafiart1ev1aaatCvAUfKttLearuWrP9MDH5MBPbIqV92AaeXatLxBI9gBaebbnrfifHhDYfgasaacH8akY=wiFfYdH8Gipec8Eeeu0xXdbba9frFj0=OqFfea0dXdd9vqai=hGuQ8kuc9pgc9s8qqaq=dirpe0xb9q8qiLsFr0=vr0=vr0dc8meaabaqaciaacaGaaeqabaqabeGadaaakeaacqWGMbGzdaWgaaWcbaGaemyAaK2aaSbaaWqaaiabdQgaQbqabaaaleqaaaaa@311D@ ) is a prefix, suffix or even a proper factor of the pseudo-exon fij
 MathType@MTEF@5@5@+=feaafiart1ev1aaatCvAUfKttLearuWrP9MDH5MBPbIqV92AaeXatLxBI9gBaebbnrfifHhDYfgasaacH8akY=wiFfYdH8Gipec8Eeeu0xXdbba9frFj0=OqFfea0dXdd9vqai=hGuQ8kuc9pgc9s8qqaq=dirpe0xb9q8qiLsFr0=vr0=vr0dc8meaabaqaciaacaGaaeqabaqabeGadaaakeaacqWGMbGzdaWgaaWcbaGaemyAaK2aaSbaaWqaaiabdQgaQbqabaaaleqaaaaa@311D@.

An EST factor *s*_*j*_, corresponding to a gene exon *factor*(fij
 MathType@MTEF@5@5@+=feaafiart1ev1aaatCvAUfKttLearuWrP9MDH5MBPbIqV92AaeXatLxBI9gBaebbnrfifHhDYfgasaacH8akY=wiFfYdH8Gipec8Eeeu0xXdbba9frFj0=OqFfea0dXdd9vqai=hGuQ8kuc9pgc9s8qqaq=dirpe0xb9q8qiLsFr0=vr0=vr0dc8meaabaqaciaacaGaaeqabaqabeGadaaakeaacqWGMbGzdaWgaaWcbaGaemyAaK2aaSbaaWqaaiabdQgaQbqabaaaleqaaaaa@311D@) is defined as internal or external depending on whether both donor and acceptor splices are or are not present, respectively at its genome boundaries after alignment. Thus, factors *s*_1_, *s*_*k *_of the EST factorization <*s*_1_, *s*_2_, ..., *s*_*k*_> are called *external factors *while *s*_2_, ..., *s*_*k*-1 _are called *internal factors*.

In other words, an EST factorization is induced by an alignment of the EST to exons of the genomic sequence. Each EST factor must correspond or align to an exon. The external EST factors can correspond to a fragment (a prefix or a suffix) of the relative exons.

By using the above stated notions, the MEFC problem is defined as follows. The instance of the problem consists of a genomic sequence *G *and a set of EST sequences (transcripts), while a solution consists of one gene-factorization *G*_*E *_of *G *and EST factorizations that are compatible or variant compatible with *G*_*E*_. Thus an *optimal *solution in the MEFC problem (that is an optimal gene-factorization and optimal compatible EST factorizations) is the one that minimizes the number of distinct pseudo-exons in the gene-factorization of the genomic sequence.

### Generation of nearly optimal compatible genome-EST alignments

The ASPIC software implements an heuristic method for the MEFC problem stated before.

The general structure of the method consists of:

(a) an initial pre-processing of the genomic sequence,

(b) two main procedural phases applying criteria to minimize splice sites.

In the following we provide a detailed description of the method by first describing the initial pre-processing phase and then the main algorithmic steps of the two phases.

#### Pre-processing of the genomic sequence

The alignment of a single EST factor to the genomic sequence is based on the notion of a *component*: a *component *is a substring of the genomic sequence that perfectly matches a portion of an EST factor. The length of a component is a critical parameter used to accelerate the alignment of EST factors as well as for finding error-free matching regions between ESTs and the genomic sequence. Indeed, components of a given length (for example 15 bp) may have very few occurrences on a genomic sequence, thus making the process of locating EST factors very fast. For this reason, the length of a component is computed automatically as a function of the gene sequence length, but it can be also modified by the user as an input parameter. The algorithm starts with an initial pre-processing of the genomic sequence *G *that consists in building a hash-table containing all occurrences of each component in *G*. Thus a key list of components (i.e. substrings of the genome) provides the entry of a Hash Table used to speed up the alignment process of an EST factor to the genomic sequence. Since the algorithm locates the intron regions by validating the splice sites using first the *GT-AG *rule, a second hash-table for all *GT *and *AG *occurrences on the genomic sequence, is initially computed and stored.

#### Phase 1: iterative computation of all EST internal factors

The first phase is an iterative processing of each EST in the set *S *= {*S*_1_, ..., *S*_*m*_} such that the general *i *iteration produces an alignment of each EST in the set {*S*_1_, ..., *S*_*i*_} compatible with a partial gene-factorization of *G *– the generation of an EST alignment against the genomic sequence implying an EST factorization. The generic step of the iteration in our algorithm consists of finding the next factor *s*_*j *_of a partial EST factorization <*s*_2_, ..., *s*_*j*-1_> and the corresponding exon along the genomic sequence. In this phase the EST-factorization is produced using a criterion, called *concatenating exons*, to minimize the number of exons. This criterion consists of concatenating two or more consecutive EST factors into a unique exon whenever a true exon may have been over factorized because of repeated regions in the genomic sequence (see as an example Figure 1).

More precisely, given the alignment of the internal factors <*s*_2_, ..., *s*_*i*_> of an EST, then the genomic alignment of a new EST factor *s*_*i*+1 _is computed in four main steps.

**Figure 2:**
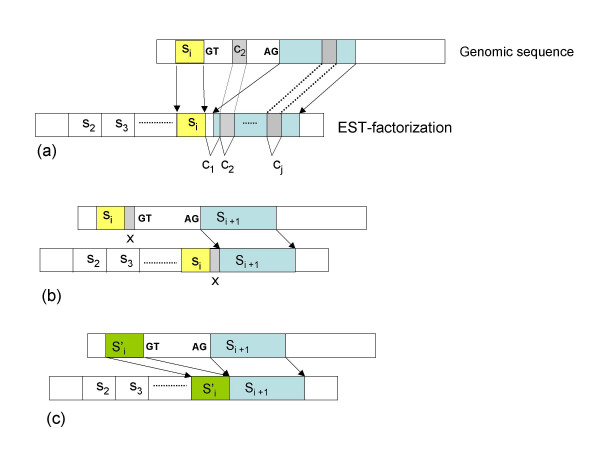
Location of a new EST internal factor *s*_*i*+1 _given previous computed factors *s*_2_, ..., *s*_*i*_. (a) Consecutive sequence components *c*_1 _... *c*_*j *_are tested to find the first one that allows the identification of a genomic region that optimally aligns factor *s*_*i*+1 _(i.e. alignment extension on one or both sides of the component): such a region is determined in (b) by the component *c*_*j*_. Figure (b) shows that some intervening positions (sequence x) may occur between factor *s*_*i *_and *s*_*i*+1_. Indeed, in this case the placement of *s*_*i*+1 _gives the correct right end of previous factor *s*_*i*_, since the larger factor  inducing canonical splice sites on the genomic sequence can be optimally aligned before *s*_*i*+1 _thus leading to an optimal location of both *s*_*i *_and *s*_*i*+1_.

In step (1) the EST suffix to be aligned after factor *s*_*i *_is divided into consecutive strings *x*_1_, *x*_2_, ..., *x*_*n *_of the predefined length of a component. Indeed, the first possible genomic location of EST factor *s*_*i*+1 _is determined by finding the leftmost string *x*_*j *_of the EST suffix that is a component and allows the optimal alignment of the entire EST factor s_*i*+*i *_(see Fig. 2(a), (b)). In step (2), for each occurrence of a component *x*_*j *_along the genomic sequence, a genomic region of maximal length containing *x*_*j *_is optimally aligned in linear time and space (using the edit-distance within a Kband [26]) to the new EST factor *s*_*i*+1_, until a compatible alignment is found (i.e. few errors are allowed and possibly canonical splice sites are located). Note that step two may fail to compute the new EST factor *s*_*i*+1_, whenever the previous EST internal factors <*s*_2_, ..., *s*_*i*_> do not allow the generation of an EST-factorization compatible with the partially computed gene-factorization. Indeed, some EST factors may have been incorrectly computed because of a wrong alignment of the EST sequence. *Backtracking *allows the relocation of exons. This consists of trying alternative occurrences in the genomic sequence of components of previous factors starting from *s*_*i *_up to *s*_2_.

Once the location of factor *s*_*i*+1 _is determined, the *concatenating exon *criterion is applied in step (3) which consists of testing whether one or more consecutive EST factors preceding factor *s*_*i*+1 _can be concatenated to *s*_*i*+1 _to obtain a unique factor *s *such that it optimally aligns to the genomic sequence. In this case, *s *replaces a list of consecutive EST factors, thus minimizing the number of exonic regions in the gene-factorization (see for example exons *AB *and *DE *in Figure 1(C) produced by the application of concatenating exon criterion to *A *and *B *first, and then to *D *and *E*). Clearly, after the minimization, the new EST factor *s*_*i*+1 _as well as previous factor *s*_*i *_are redefined so that the EST alignments define a smaller number of exons.

Finally, in step (4), a dynamic programming (DP) algorithm is used to refine the intron boundaries between the defined EST factors *s*_*i *_and *s*_*i*+1_. This crucial step of the algorithm is detailed in the next section *Refining intron boundaries*.

Observe that the location of a new EST factor *s*_*i*+1 _is based on the use of a single component (that is a perfect matching region) and that such a component is located on the factor by testing consecutive positions in the EST suffix after factor *s*_*i*_. This approach may imply that several positions after the right end of EST factor *s*_*i *_are skipped before placing the left end of the new factor *s*_*i*+1_. Indeed, in such cases the placement of factor *s*_*i*+1 _may imply an extension (or a reduction) of the right end of previous factor *s*_*i *_thus optimizing exon definition (see Fig. 2(c)). This strategy makes the alignment process more flexible and faster with reference to other approaches (such as BLAT [19]) that apply strict matching criteria.

Indeed a feature of ASPIC alignment algorithm is that it allows a fast exact location of the alignment regions of EST factors without necessarily comparing all EST sequences against large portions of the genomic sequence. Consequently, ASPIC also allows EST alignment in the presence of a relatively high number of errors that are located in specific regions. Moreover, even though the alignment process relies on dynamic programming (DP) it turns out to be very fast in most of the cases, as indeed DP is only applied to short portions of the EST and genome sequence.

#### Phase 2: refining internal factors and placing external factors

**Figure 3:**
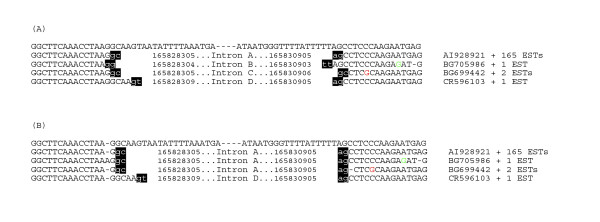
Example of intron detection in the human ATP1B1 (UG:Hs.291196) gene without (A) or with (B) the refinement of exon-intron boundaries. The first row shows the genomic sequence aligned to the EST sequences (below). In (A) four different introns are detected (A, B, C, D) that can be merged to only two (A, D) in B. Absolute coordinate (NCBI 35 assembly) are shown for each intron and acceptor/donor splice sites are in black-background.

This phase of the algorithm completes the computation of all EST factorizations (i.e. EST alignments) by first correcting all internal EST factors pre-computed in the first phase in order to make all factorizations compatible with the same gene-factorization *G*_*E *_of *G *minimizing the number of splice sites. More precisely, the minimization relies on the use of a criterion called *merging splice sites*. Merging splice sites consists of comparing computed exons *x *and *y *supported by EST factors to reduce the intron boundary of *x *to the one of *y *or vice versa, whenever they differ at only a few positions, likely because of sequencing errors in the EST factors (see an example in Fig. 3). Clearly, this step may avoid over prediction of splice sites due to the erroneous location of intron boundaries because of sequencing errors. This criterion is also implemented to allow the detection of possibly true splice variants determined by competing 3' or 5' junctions induced by few bases (two bases or more).

Finally, after the localization of EST internal factors, all EST external factors are computed. The *concatenating exons *and *merging splice sites *criteria are used again since errors in EST sequences are more prevalent in terminal regions, which may be as short as few bases – thus permitting several alternative alignments. The procedure that finds external EST factors tries to align the EST leftmost (or rightmost) factor as a suffix (or a prefix) of some previously computed exon. If that is not possible, the factor is placed in a new location in correspondence with a *GT *(or *AG*) pattern and then the DP algorithm is used again to refine intron boundaries.

### Refining exon-intron boundaries

Because of sequence repeats and sequencing errors in ESTs, the exact location of splice junctions is a critical issue [27]. Our method combines different strategies to evaluate and hence improve the quality of splice data produced. These are listed below:

1. *Finding intron boundaries via dynamic programming*. A first criterion used to find the exact location of intron boundaries is the evaluation of alignment quality. We have designed an algorithm, based on dynamic programming (DP), to produce optimal alignments of regions close to splice sites. It computes the genomic alignment of a suffix *w *and a prefix *y *of two consecutive EST factors, *s*_*i *_and *s*_*i*+1_, in order to locate in the genomic sequence the optimal position for a *single large gap *corresponding to the intron region. This gap may not be delimited by canonical splice sites following the *GT *– *AG *rule, which is recognized as a basic one for the validation of splice sites, as more than 98.7% annotated splice sites in GenBank are canonical in this respect [18]. Indeed, there may be different optimal alignments leaving a gap with the same error rate. Thus a second important algorithmic step is applied by ASPIC to locate splice sites.

2. *Canonical patterns and weight matrices*. Whenever the optimal alignment computed via DP does not lead to canonical splice junctions, then the algorithm looks for alternative alignments with the same error rate with preference for the couple of splice boundaries more frequently represented in the weight matrix provided in [18] (see Table 2 in [18]). If different alignments of the same quality (i.e. number of errors) are possible near intron boundaries, the choice of the alignment is done by using the weight matrix. For example, the base-pairs GC-AG are selected before the pair AT-AG if compatible with an alignment of splice sites leaving the same number of errors, as GC-AG is more frequent than AT-AG in the weight matrix. Clearly, an high quality alignment may also lead to the acceptance of splice sites with null frequency in [18] matrices.

**Table 2:** Splice sites in known and novel ASPIC-predicted introns

Splice Site	Known introns		Novel introns	
	*N*	*%*	*N*	*%*

GT-AG	897	98.14	57	60.64
GC-AG	8	0.77	15	15.96
GT-other	3	0.33	5	5.10
other-AG	4	0.44	13	13.27
other-other	3	0.33	4	4.08
*Total*	915		94	

Number (N) and percentage (%) of splice site types in known and novel ASPIC-predicted introns.

Actually, the presence of sequencing errors may often complicate the location of the correct splice sites junctions. For these reasons, the use of agreement criteria among EST alignments turns out to be crucial in many practical cases to detect highly confirmed splice junctions and thus to correct ambiguous alignments.

Moreover, in order to evaluate the quality of splice sites we annotate each detected splice site, either donor or acceptor, with a consensus sequence and a score: the score derives from the formula and tabular nucleotide frequencies reported in [23]. Indeed, conserved splice sequences provide further evidence for splice junctions.

3. *Congruence of ESTs on the location of splice sites*. Since the merging splice site criterion discussed in the previous section is based on a combined analysis of all EST factorizations, it is crucial also for validating intron boundaries. Indeed, by comparing EST factors it is possible to discover sequencing errors in ESTs that show that some intron boundaries must be considered as coincident if few errors are tolerated (typically at most one error for each splice site) or even by shifting the location of canonical splice sites. For example, in many cases the GT-AG rule may be applied to locate an EST factor boundary in two very close locations of the genomic sequence, thus making the choice of the alignment near intron boundaries for a single EST difficult. In these cases, an independent EST alignment does not allow the determination of the EST splice sites, while the presence of other EST factorizations having a better quality alignment to the genomic sequence may solve the aforementioned dilemma because of the common compatibility to the exon-intron structure. This situation is detailed in the example shown in Fig. 3.

4. *Filtering artifacts and locating gene strand*. Our implementation has automatic procedures to locate the strand from which each EST originates (independently from the cluster annotation) and a filtering of possible artifacts and polyA ends. Moreover, EST alignments of poor quality are filtered out based on several criteria, including a percentage of sequence identity below the fixed cutoff.

As an example, Figure 3 reports the optimal alignments of ESTs close to intron boundaries illustrating the need for specific criteria to locate all plausible intron boundaries. The basic criterion is the congruence of ESTs near splice sites, combined with the use of known frequencies of splice patterns (see [18]). ATP1B1 introns B and C (Fig. 3A) can disappear by merging them to intron A (confirmed by a large number of ESTs) after the introduction of a A-insertion or of a C-deletion in the relative alignments. On the other hand, intron D is likely to represent a genuine variant. In all these cases it is likely that the relevant EST sequences are not correct due to a typical base miscalling in single-read automatic sequencing, i.e. AAA instead of AA for BG705986 and C instead of CC for BG699442.

### Clustering ESTs by common splice sites

For each splice site predicted, ASPIC provides the list of ESTs supporting such splice sites, thus allowing the evaluation of the quality of the prediction in terms of number of ESTs confirming it. Moreover, this step allows the grouping of ESTs that strongly support a common transcript (by sharing the same sequence of splice sites).

### Minimal set of full-length transcript isoforms

Since a feature of ASPIC is to report splice sites and corresponding factorization into genomic exons for each EST *(EST-exon-factorization *in our terminology), we have designed and implemented in the module *Transview *of ASPIC an efficient algorithm that combines EST-exon-factorization data into a set of minimal full-length transcripts that are supported by the evidence, i.e. by the set of available ESTs. Our algorithm is based on the use of directed acyclic graphs (DAG): nodes of the graph are EST-exon-factorizations, while edges connect nodes (sequences) that are related by a binary relation among EST-exon-factorization (*extension*). Paths in the graph represent possible full-length transcripts. Various methods based on graphs have been reported to predict transcripts from ESTs such as in [28,10] and [17]: our method is different from those approaches in the construction of the graph as well as in the way the graph is visited to report full-length transcripts. In contrast to graph based approaches proposed in [17] or [11] where nodes are exons or nucleotide sequences, our approach uses a reduced graph and an efficient visiting process that allows the reporting of all plausible paths, without requiring a trimming phase as in [17] to remove redundant models. Indeed, our algorithm aims to reduce over predictions or false positives as well as to reduce the execution time required by the construction of a potentially exponential number of paths (putative full-length transcripts) in the graph. Moreover, the construction of the graph in our model is guided by input parameters that allows the user to specify the quality of predicted full-length transcript with respect to the set of transcripts supporting them.

*Transview *provides a visualization of full-length isoforms and for each predicted full-transcript their composition in terms of the ESTs that support the full-transcript. Details on the algorithm will be discussed elsewhere.

## Results

The capability of ASPIC method to computationally produce high quality gene predictions has been tested by performing two types of experiments. A first experiment consisted in comparing ASPIC data with data available from other database sources that collect intron-exon data obtained through computational as well as experimental methods. This first experiment shows the ability of ASPIC in predicting novel splice variants as well as in detecting good quality splice sites confirmed by other sources. In order to assess the quality and reliability of novel predictions, a second experiment has been carried out: this one consisted in comparing ASPIC data with those produced within the ENCODE project [29] aimed at providing a reliable annotation of 1% of the human genome. In particular, we investigated the occurrence of false positives in ASPIC-predicted introns as determined by RT-PCR analysis for 22 genes located in 13 Encode regions.

### Comparing ASPIC with other similar tools

**Figure 4:**
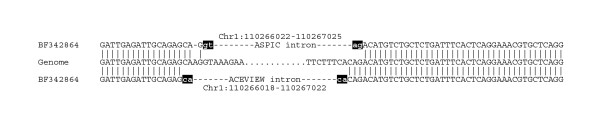
Example of intron boundaries detected for the human AHCYL1 gene by AceView and ASPIC. The hypothetical novel intron predicted by AceView (July 2003 release) with non-canonical splices can be reduced to a known intron by a single A-insertion. Intron coordinates are referred to Ensembl release 26.35.1.

The ASPIC method has been tested on a sample of 64 genes randomly chosen from the human Chromosome 1. Results are summarized in Table 1 where they are also compared with those obtained by other publicly available resources. A total of 1009 introns were predicted by ASPIC as compared to 753 by ASAP, 495 by ASD and 1194 by AceView. ASPIC predicted 95.7%, 93.1% and 75.8% of introns predicted by ASAP, ASD and AceView, respectively. In general, predicted introns were well supported by genome-transcript alignments with 28.3 ESTs supporting each splice site on average. Missing introns may derive from additional ESTs not present in the UNIGENE cluster used by ASPIC or by the stringent parameter thresholds adopted in ASPIC to consider an intron prediction reliable. The large number of additional introns detected by AceView, but not by other resources, are partly due to the wrong selection – in some cases – of the genomic region to be considered for the analysis. For example, AceView predicts 45 introns in the gene AMPD1 w.r.t. the 14 introns predicted by ASPIC (13 in ASAP). In this case the genome region selected by AceView encompasses 113 kb covering AMPD1 and two additional genes. A similar problem can be observed with several other genes where the number of AceView introns is remarkably higher than that detected from other resources (e.g. ADAM15, AKR7A2, ARNT, ARPC5, ATAD3A, etc.). Also, AceView intron over-prediction is likely due to the use of less stringent parameters in genome-transcript alignments, as in the example shown in Fig. 4.

However, ASPIC detected a total of 94 novel introns, each confirmed by 2.18 ESTs on average. It is interesting to note that our data show a higher occurrence of non-canonical splice sites with respect to previous estimates [30]. Table 2 shows splice sites for known and novel ASPIC predicted introns. These data are not unexpected as previous estimates did not consider most of the splicing variants of annotated genes. While some of the predicted introns may simply be artifactual it is likely that rarer splicing isoforms involve a higher proportion of non-canonical splice sites. Another striking observation from our analysis is that 62/64 genes (97%) show alternative splicing with an average of 11.9 transcripts/gene, a value similar to that from AceView data (see Table 1) but significantly higher than 2.3 and 5.1 estimated by ASAP and ASD repectively. It is worth mentioning that data reported by ASAP are not updated w.r.t. the latest Unigene/genome data and several genes (28/64) were not annotated in ASD. It should be considered that Unigene clusters are enlarging at a great rate and genomic sequences are also continuously updated. To address this problem ASPIC data are stored in a dynamic database. The relevant data for each gene query are stored in the ASPIC database so that if another user does a similar query the results are immediately available without carrying out a new analysis. However, the user can choose to overwrite stored data with updated genome and transcript data directly extracted from Ensembl and Unigene databases. The new data remain stored in the ASPIC database until a new overwrite request for the same gene query is made.

### False positive incidence of ASPIC introns

In order to compare the false positive rate of introns predicted by ASPIC and other methods we analyzed the GENCODE experimental verification of computationally predicted introns for a set of 22 genes in 13 Encode regions (see the GENCODE annotations in the Additional file 2). Of the total 44 introns not supported by RT-PCR experiments (labeled RT_negative) ASPIC supported only 12/44 whereas AceView supported 41/44 (Table 3). Interestingly, 7/12 ASPIC introns were supported by more than 2 ESTs, also showing high-scoring slice patterns (see Additional file 3). This finding suggests possible leakages in experimental validations carried out within the Encode project.

**Table 3:** RT-negative introns supported by ASPIC

Encode Region	Gene	Intron position			Prediction Method
		*Chr*	*Start*	*End*	

ENm004	SLC5A1	22	30779886	30787475	ASPIC (3), ECgene, acembly
ENm004	PISD	22	30350425	30351061	ASPIC (1), ensEstGene
ENm004	PISD	22	30337622	30338657	acembly
ENm004	PISD	22	30346557	30351299	acembly
ENm004	PISD	22	30365972	30366216	acembly
ENm004	RFPL3	22	31075439	31078694	acembly
ENm004	SYN3	22	31727364	31734939	acembly
ENm004	TIMP3	22	31521971	31522263	acembly
ENm004	TIMP3	22	31521971	31522271	acembly
ENr223	MTO1	6	74249065	74253041	ASPIC (6), ECgene, acembly
ENr223	MTO1	6	74253206	74258677	ASPIC (5), ECgene, acembly
ENr231	PSMD4	1	148044771	148047709	ASPIC (2), ECgene, acembly
ENr231	PIP5K1A	1	148035078	148039516	acembly
ENr231	PIP5K1A	1	148035192	148035350	acembly
ENr231	PSMB4	1	148187431	148194586	acembly
ENr231	PSMD4	1	148040228	148047741	acembly
ENr231	PSMD4	1	148040358	148044611	acembly
ENr231	PSMD4	1	148044684	148047709	acembly
ENr231	PSMD4	1	148046796	148047709	acembly
ENr231	SNX27	1	148423496	148424527	acembly
ENr231	TUFT1	1	148350164	148356015	acembly
ENr231	TUFT1	1	148356492	148372163	acembly
ENr232	CRAT	9	128949911	128950731	ASPIC (1), acembly, softberryGene
ENr232	PPP2R4	9	128953625	128962345	ASPIC (1), ECgene
ENr232	PPP2R4	9	128952305	128953168	acembly
ENr232	PPP2R4	9	128952336	128953268	acembly
ENr232	PPP2R4	9	128952981	128953304	acembly
ENr232	PPP2R4	9	128953105	128953150	acembly
ENr232	SH3GLB2	9	128835746	128849868	acembly
ENr232	SH3GLB2	9	128860722	128862923	acembly
ENr323	LACE1	6	108794230	108829892	ASPIC (5), sgpGene
ENr323	LACE1	6	108747689	108751721	acembly
ENr323	SNX3	6	108688727	108690771	acembly
ENr333	RNPC2	20	33764744	33765167	ASPIC (1), ECgene, acembly
ENr333	RNPC2	20	33786418	33787848	ASPIC (1), ECgene
ENr333	CEP2	20	33527835	33529106	acembly
ENr333	CEP2	20	33554537	33568378	acembly
ENr333	CEP2	20	33561298	33568224	acembly
ENr333	ITGB4BP	20	33335958	33343927	acembly
ENr333	RNPC2	20	33776436	33777255	acembly
ENr333	RNPC2	20	33780699	33780701	acembly
ENr333	SDBCAG84	20	33585738	33593682	acembly
ENr334	TFEB	6	41766952	41811861	ASPIC (5), ECgene, acembly
ENr334	TFEB	6	41766952	41799176	ASPIC (3), ECgene, acembly

List of computationally predicted introns of 22 genes contained in 13 Encode regions (see the GENCODE annotations in the Additional file 2) but not validated by RT-PCR analysis. For each intron are shown the Encode region, the gene ID, location (NCBI 35 assembly) and prediction methods (Acembly/AceView, ; ECgene [17]; ensEstGene [28]; softberryGene [35]. For ASPIC predictions the number of supporting in shown in the brackets.

### The ASPIC Web Resource

The ASPIC program can be accessed online at: . ASPIC standard input data consist of a genomic sequence and a set of transcripts. Such data are acquired either automatically or by uploading files specified by the user. In the first case, a basic form permits the input of an official HUGO gene name for the genomic sequence (e.g. ABCB10, HUGO names are permitted only for human genes) and/or a Unigene cluster identifier (e.g. Hs.1710). EST clusters are automatically retrieved from Unigene, while genome sequences are retrieved by using the API provided from Ensembl. All results presented here are based on one of the latest releases (September 2004 Ensembl API release .25 and 2004 Unigene database release).

The automatic acquisition of clusters is allowed for human and every other organism whose data may be acquired from the Ensembl database. A specific upload function allows the user to query ASPIC processing of arbitrary genomic sequences and transcript data in FASTA format.

An advanced search form allows the user to run the ASPIC program by specifying basic parameters used to produce compatible EST alignments.

We have tested our method using standard parameters suggested by experimental analysis of real data. For example, we choose a minimum exon length of 15 nt. The component length for building hash tables is computed by using a formula that relates the minimum exon length to the component length in such a way that the existence of an error-free substring in an EST factor is guaranteed.

**Figure 5:**
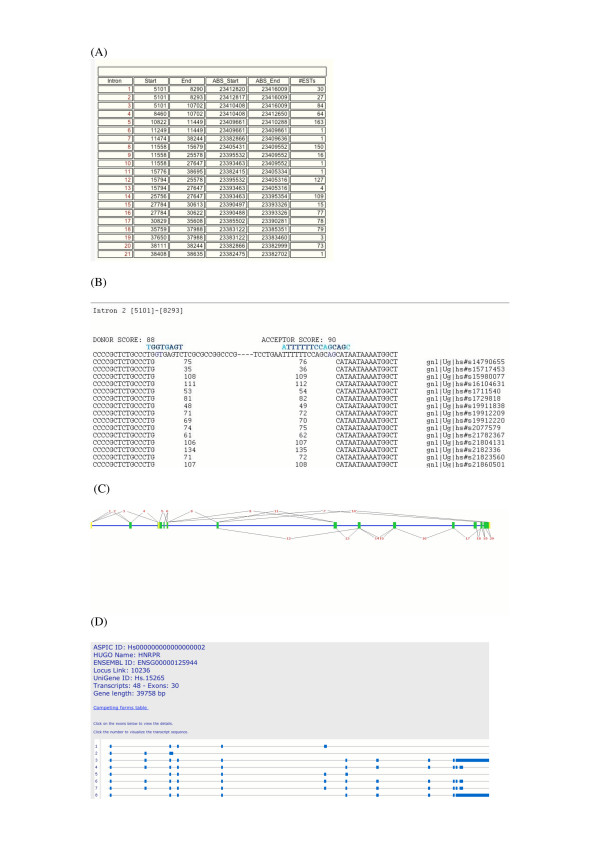
Snapshot of the ASPIC output for the gene HNRPR (human chromosome 1). The Table View (A) lists all detected introns, their coordinates and the number of supporting ESTs. The Alignment View (B) shows the alignment between genomic and EST sequences around splice sites. The Graphical View (C) provides a general scheme of the splicing pattern. The Transcript View (D) shows the minumum set of different transcripts compatible with the detected splicing patterns.

ASPIC outputs a complete description of each EST exon-factorization, with a view of the alignment to the genomic sequence, as well as a tabulated view of splice sites. The program provides an output file that contains detailed information about all EST exon-factorizations. This file is also processed by Perl scripts in order to produce and make available to the user from the ASPIC web site: i) a table view listing all detected introns; ii) a graphical view showing the general exon-intron arrangement of the queried gene; and iii) a transcript view showing all non-mergeable transcript models compatible with detected introns. In particular, the table reports the relative and absolute coordinates of each detected intron derived from the genomic sequence and genome build considered, respectively, as well as the number of confirming ESTs. Absolute coordinates, not provided by other resources, are particularly useful for the comparison of intron coordinates for a gene to those annotated in genome browsers. The main graphical view is a visualization of the intron structure of the genomic sequence derived from the tabulated data. Such a graphical view also provides links to a visualization of the alignment of the 15 base pairs of EST sequences closest to intron boundaries. Figure 5 shows an example of the table, the graphical and the transcript view.

#### ASPIC Execution time

The performance of ASPIC has been evaluated on a Pentium IV class PC, with 256 MB of main memory running the Linux operating system.

The processing time for a single EST varied from 0.007 sec cpu time to a maximum of 2.5 sec cpu time, where the gene length varied from 5014 bp to 287011 bp, requiring on average around 71 seconds cpu time per gene. Thus ASPIC can process about 5000 ESTs in about half an hour of cpu time (against the four hours required in [16]).

#### Experimental results: WEB-sources

The comparison of ASPIC data with other sources of splice sites has been carried out by accessing available databases from the web at the following sites: ASD [31], ASAP [32], Acembly [33].

## Conclusion

The ASPIC algorithm implements a novel methodology that optimizes the overall compatibility between genomic and transcript sequences to detect splice sites – thus minimizing mispredictions due to repetitive sequences or sequence errors in the ESTs. It does not impose constraints on the splice boundaries (i.e. strict observance of the GT-AG rule) but in case of equally likely alternative alignments adjusts splice boundaries to those observed to occur more frequently in known genes [18]. Hence, it is able to detect non-canonical splice boundaries such as those of U12-dependent introns [34] in the presence of suitable supporting transcripts (see Additional file 3). Finally, ASPIC allows the user to carry out splicing predictions on a wide range of species as well as on user-submitted genome and transcript sequences.

## Availability and requirements

The ASPIC web tool is available to scientists wishing to use it at . To submit a query to ASPIC the user needs to fill a form specifying the organism, the gene ID (Ensembl or HUGO), the Unigene cluster ID (optional) and providing an email address. The request is processed by the ASPIC software and when the results are available an email is automatically sent back to the address specified by the user, providing a link to processed data.

ASPIC collects all the results of submitted queries in a dynamic database.

Project name: ASPic Alternative Splicing Prediction

Project home page: 

Programming language: C

Operating system: Debian GNU/Linux 3.1, kernel 2.6.8

Other requirements: Apache 1.3, Perl 5.8.4, Php 4.3.10, MySQL 4.1, gcc 3.3.5

## Authors' contributions

GP conceived the study. PB and RR designed the algorithms and the general ASPIC method. RR implemented the method, realized the web resources and performed the experimental analysis. All authors participated in the design of the ASPIC tool and the experimental study. All authors have contributed in drafting the article.

## Supplementary Material

Additional File 1 Splicing site prediction with and without the optimization strategy. Click here for file

Additional File 2 Gencode annotation of 13 Encode regions. Click here for file

Additional File 3 RT-negative introns detected by ASPIC. Click here for file

Additional File 4 U12 dependent introns detected by ASPIC. Click here for file
