# Supplementary material for: ASPIC: a novel method to predict the exon-intron structure of a gene that is optimally compatible to a set of transcript sequences
Source: BMC Bioinformatics. 2005 Oct 5;6:244. doi: 10.1186/1471-2105-6-244 (PMC1276783; doi:10.1186/1471-2105-6-244)
Supplement: Additional File 1 — Splicing site prediction with and without the optimization strategy. [file 1471-2105-6-244-S1.pdf]

```
 (A) Without Optimization

AW874468         180 gtcattttctttaactttgcaaactggtcttcctgctggcacatactgtg
                     |.||||||||||||||||||||||||||||||||||||||||||||||||
HsChr1     103833954 gacattttctttaactttgcaaactggtcttcctgctggcacatactgtg

AW874468             atgtcatttttggagataaaattaatggcaattgcacaggcattaaaatt
                     |||||||||.||||||||||||||||||||||||||||||||||||||||.
HsChr1               atgtcatttctggagataaaattaatggcaattgcacaggcattaaaatc

AW874468             tacgtttctgatgatggcaaagctcattttctattag          95
                     |||||||||||.||||||||||||||||||||||||
HsChr1               tacgtttctgacgatggcaaagctcattttctatta    103834091

                    <GTxxxxxxxxx...xxxxxxxxxxxxxAG>

AW874468          94 taactctgctgaagatccatttattgcaattcatgctgaatttaaattgt
                     |||||||||||||||||||||||||||||||||||||||||.||||||||
HsChr1     103880338 taactctgctgaagatccatttattgcaattcatgctgaatctaaattgt

AW874468             aaaatttaaaattaaatgcatgtcctc          18
                     |||||||||||||||||||||||||||
HsChr1               aaaatttaaaattaaatgcatgtcctc   103880414


(B) With Optimization

AW874468         180 gtcattttctttaactttgcaaactggtcttcctgctggcacatactgtg
                     |||||||.||||||||.|||||||||||||||||||||||||||||||||
HsChr1     103880200 gtcatttactttaactgtgcaaactggtcttcctgctggcacatactgtg

AW874468             atgtcatttttggagataaaattaatggcaattgcacaggcattaaaatt
                     |||||||||.||||||||||||||||||||||||||||||||||||||||
HsChr1               atgtcatttctggagataaaattaatggcaattgcacaggcattaaaatt

AW874468             tacgtttctgatgatggcaaagctcattttctattagtaactctgctga
                     |||||||||||||||||||||||||||||||||||||||||||||||||
HsChr1               tacgtttctgatgatggcaaagctcattttctattagtaactctgctga

AW874468             agatccatttattgcaattcatgctgaatttaaattgtaaaatttaaaat
                     |||||||||||||||||||||||||||||.||||||||||||||||||||
HsChr1               agatccatttattgcaattcatgctgaatctaaattgtaaaatttaaaat

AW874468             taaatgcatgtcct          18
                     ||||||||||||||
HsChr1               taaatgcatgtcct   103880414
```

Additional File 1. Exon-factorization into 2 or 1 exons of the EST AW874468 (S1938294) (*S*) (Unigene cluster Hs.514463, build #176) related to the gene AMY2A (*G*) on Chromosome 1. The EST sequence *S* agrees with the genomic sequence *G* in two possible ways: either *S* is factorized into 2 exons of *G*, that is $S = \langle C_1, C_2 \rangle$ or into 1 exon of *G*, that is $S = \langle C \rangle$ where $C = C_1 C_2$. Indeed, *G* contains two distinct ”identical” regions that align to factor *C* of EST *S*, where *C* is about 200 bp long The alignment of the same region of the EST is reported without the application of optimization criteria (2 exons) (A) and using the minimization criteria (1 exon) (B). EST and Chromosome coordinates (Ensembl release 26.35.1) are reported at start and end alignment positions.
