# Supplementary material for: ASPIC: a novel method to predict the exon-intron structure of a gene that is optimally compatible to a set of transcript sequences
Source: BMC Bioinformatics. 2005 Oct 5;6:244. doi: 10.1186/1471-2105-6-244 (PMC1276783; doi:10.1186/1471-2105-6-244)
Supplement: Additional File 3 — RT-negative introns detected by ASPIC. [file 1471-2105-6-244-S3.pdf]

Additional File 3. Alignment between genomic and transcript sequences at level of ASPIC introns supported by ≥2 ESTs not experimentally validated by RT-PCR. For each intron, the Encode region, the gene ID, the location and the Unigene ID are shown.

1) ENr223: MTO1 chr6: 74249065-74253041 (UG:Hs.347614)

```
DONOR SCORE: 82                                ACCEPTOR SCORE: 80
             AGGTATGC                            CACAAATTTTCCAGG
 AAGTCTGCCTGTCAGGTATGCATTTTTAATATAGA----AAAATACACAAATTTTCCAGGCGTGGTGGCAGACA
 AAGTCCGCCTGTCAG       232                          233       GCGTGGTGGCAGACA       AW840355
 AAGTCTGCCTGTCAG       224                          225       GCGTGGTGGCAGACA       AW840425
 AAGTCTGCCTGTCAG       225                          226       GCGTGGTGGCAGACA       AW840426
 AAGTCTGCCTGTCAG       310                          311       GCGTGGTGGCAGACA       AW840506
 AAGTCTGCCTGTCAG       285                          286       GCGTGGTGGCAGACA       BG877984
 AAGTCCGCCTGTCAG       284                          285       GCGTGGTGGCAGACA       BG877995
```

2) ENr223: MTO1 chr6: 74253206-74258677 (UG:Hs.347614)

```
 DONOR SCORE: 69                               ACCEPTOR SCORE: 86
             CAGTATGT                            TCATATATTTGCAGA
 GAAAAGACTCATACAGTATGTGTTTTTGTGACTGG----AAACTTTCATATATTTGCAGAGCTCTCGATGTTCT
 GAAAAGACTCATACA       396                          397       AGCTCTCGATGTTCT       AW840355
 GAAAAGACTCATACA       389                          390       AGCTCTCGATGTTCT       AW840425
 GAAAAGACTCATACA       389                          390       AGCTCTCGATGTTCT       AW840426
 GAAAAGACTCATACA       449                          450       AGCTCTCGATGTTCT       BG877984
 GAAAAGACTCATACA       448                          449       AGCTCTCGATGTTCT       BG877995
```

3) ENr323: LACE1 chr6: 108794230-108829892 (UG: Hs.259666)

```
 DONOR SCORE: 79                               ACCEPTOR SCORE: 96
             AGGTAAAA                            TTTTTTTCTTTCAGA
 ACAGGCCACCGGAAGGTAAAAACAAACATTGTGCT----ATATCATTTTTTTCTTTCAGATCTCTATAAAAATG
 ACAGGCCACCGGAAG       878                          879       ATCTCTATAAAAATG       BX395362
 ACAGGCCACCGGAAG       497                          498       ATCTCTATAAAAATG       BE258784
 ACAGGCCACCGGAAG       934                          935       ATCTCTATAAAAATG       BC018445
 ACAGGCCACCGGAAG       934                          935       ATCTCTATAAAAATG       NM_145315
 ACAGGCCACCGGAAG       934                          935       ATCTCTATAAAAATG       AF520418
```

4) ENr334: TFEB chr6: 41766952-41811861 (UG: Hs.485360)

DONOR SCORE: 88 ACCEPTOR SCORE: 90

TGGTGAGT **CTTCTCATCCACAGG**

AGCAAGCTCAGGCTGGTGAGTGGGGCGTGAATGTA----CTGCCCCTTCTCATCCACAGGGAGCCAGCGCCGGC

AGCAAGCTCAGGCTG	90	91	GGAGCCAGCGCCGGC	BX385749
AGCCAGCTCAGGCTG	78	79	GGAGCCAGCGCCGGC	BX339484
AGCAAGCTCAGGCTG	78	79	GGAGCCAGCGCCGGC	BX381616
AGCAAGCTCAGGCTG	114	115	GGAGCCAGCGCCGGC	AJ608786
AGCAAGCTCAGGCTG	77	78	GGAGCCAGCGCCGGC	BP321194
AGCAAGCTCAGGCTG	89	90	GGAGCCAGCGCCGGC	BX333562

5) ENr334: TFEB chr6: 41766952-41799176 (UG: Hs.485360)

DONOR SCORE: 82 ACCEPTOR SCORE: 90

AGGTATGA **CTTCTCATCCACAGG**

GCTGCCCTCTCGAAGGTATGAGAGCACACGCGTGC----CTGCCCCTTCTCATCCACAGGGAGCCAGCGCCGGC

GCTGCCCTCTCGAAG	266	267	GGAGCCAGCGCCGGC	AJ608791
GGCGCCCTCTCGAAG	83	84	GGAGCCAGCGCCGGC	BI757401
GCTGCCCTCTCGAAG	58	59	GGAGCCAGCGCCGGC	BI911820

6) ENm004: SLC5A1 chr22: 30779886-30787475 (UG: Hs.1964)

DONOR SCORE: 92 ACCEPTOR SCORE: 93

AGGTAAGA **TTTTGCGTTTCCAGA**

CAGACAACACATGAGGTAAGAGATTGGAGGATGGA----ACTTTCTTTTGCGTTTCCAGATTGGAGCCTCCCTC

CAGACAACACATGAG	157	158	ATTGGAGCCATCCTC	R57161
CAGACAACACATGAG	174	175	ATTGGAGCCTCCCTC	AL599996
CAGACAACACATGAG	149	150	ATTGGAGCCTCCCTC	AL693702

7) ENr231: PSMD4 chr1: 148044771-148047709 (UG: Hs.505059)

DONOR SCORE: 59 ACCEPTOR SCORE: 76

AGGTTGAG **CCCTAATCTGACAGT**

TACTACCCTAGACAGGTTGAGTATTCCCTATCTGA----CTTCAACCCTAATCTGACAGTGTGGACAACAGTGA

TACTACCCTAGACAG	51	52	TGTGGACAACAGTGA	AA351224
TACTACCCTAGACAG	29	30	TGTGGACAACAGTGA	AA351328
