# Supplementary material for: ASPIC: a novel method to predict the exon-intron structure of a gene that is optimally compatible to a set of transcript sequences
Source: BMC Bioinformatics. 2005 Oct 5;6:244. doi: 10.1186/1471-2105-6-244 (PMC1276783; doi:10.1186/1471-2105-6-244)
Supplement: Additional File 4 — U12 dependent introns detected by ASPIC. [file 1471-2105-6-244-S4.pdf]

Additional File 4. ASPIC detected introns on a sample of 32 U12 introns listed in Table 5 of Levine and Durbin (2001). Genome coordinates (NCBI assembly 35), mRNA alignment, HUGO and representative mRNA ID and number of supporting transcripts are reported for each intron.

```
TAATCAAGGAAACACGTATCCTTTCAGTGTTTACC----CTTTAACCATTTTTATTCAGAGGCTCCAGCATATC
TAATCAAGGAAACAC            166673398...166678883           AGGCTCCAGCATATC    U59919   + 35 ESTs

AGAAGAAGAAAGCTGATATCCTTTTAGAGTGGCAT----ATCCTTAACAAGTTGTTTACTTGATGAAGACCCTG
AGAAGAAGAAAGCTG            166732784...166735170           TTGATGAAGACCCTG    U59919   + 18 ESTs

CAGAAATCGAAGATCATATCCTTGAACTGTGCCGG----CCGGCCCTTGACTGTGTCACATATCTACCAAGAGC
CAGAAATCGAAGATC          chr1:23465833...23469343           ATATCTACCAAGAGC    TCEA3 (AJ223473 + 31 ESTs)

AGACCCCAAAACCAGGTATTCTTTTTTGGGATGGA----TGCCAATTTTCTTCTTTCAGGAGAGACTCTGTGGA
AGACCCCAAAACCAG          chr1:23480526...23489047          GAGAGACTCTGTGGA    TCEA3 (AJ223473 + 26 ESTs)

TGATTGATAACCTCTGTATCCTTCAGACATTCAGC----CTTAACAATGGGGCCAAAAGCTGCCAGCAATCATT
TGATTGATAACCTCT          chr1:38000654...38001223          CTGCCAGCAATCATT    INPP5B (M74161   + 10 ESTs)

GAATACTGCGTCATCATATCCTTCCTGTGCTAACT----CCCTTGACTCTGAGCCGCAGGCGGTGCAAGGTGTG
GAATACTGCGTCATC          chr1:38080616...38081016          GCGGTGCAAGGTGTG    INPP5B (M74161   + 8  ESTs)

AGATTGCAAACAACAGTATCCATGCCCTTGGCCAG----CCTTGACCAGATCCCTCCAGGCTCCCAGATTCTGC
AGATTGCAAACAACA            130719136...130720647           GCTCCCAGATTCTGC    AF244931 + 61 ESTs

GGGCATCTCTAAAGTGTATCCTTTCTCTTATCCCT----CTTAACCCTGTTCACGGCAGGAAAATACTCTTCAC
GGGCATCTCTAAAGT            130713954...130715929           GAAAATACTCTTCAC    AF244931 + 37 ESTs

GTTGAAAAAATGACCATATCCTCTCTTGAAAGGAG----CCTTTACTCCTTGGCTGCACAGCTTTGTGAAGGAT
GTTGAAAAAATGACC            140886213...140887323           AGCTTTGTGAAGGAT    AF051782 + 31 ESTs

TCGCATGGAGATGGAATATCCTTTTGCTGACTAGG----AGCCTATTTCCCTTTTCCACTGACTTTAATGAAGT
TCGCATGGAGATGGA            140937343...140937975           TGACTTTAATGAAGT    AF051782 + 6  ESTs
```

```
CAACTTCCCTCGTGTGTATCCTTTCCCAGGCCTGG----TTTCCTTGACGCCGCCGCAGACTTCAGCAGTGTGG
CAACTTCCCTCGTGT             66542116...66542365            ACTTCAGCAGTGTGG    AF047338 + 31 ESTs

AGGGCGACGAGAACTGTATCCCTTTGTGGAGAGGC----CTTGACTTGAGGCCCTGCAGACATGGAGTTCCTCG
AGGGCGACGAGAACT             66536336...66536490            ACATGGAGTTCCTCG    AF047338 + 31 ESTs

GGATTCTTAATGACCGTATCCTTCAACAAACAACA----TCCTTAATGCCATCTGCTAGCTTTTTGGTCTGTTT
GGATTCTTAATGACC              5324627...5325339             CTTTTTGGTCTGTTT    AF242523 + 72 ESTs

TAACATGATTTTTCTGTATCCTTTATTAAATGGGG----TTCTTAACTTGGAACTTAAGATATCGTTACTGTCG
TAACATGATTTTTCT              5325434...5326852             ATATCGTTACTGTCG    AF242523 + 97 ESTs

GTCACTATGGTAGAGATATCCTTTGTTGTATAAGA----CTCCTTAACCTGTTGCGGACATTGACCAAATGGTG
GTCACTATGGTAGAG             21755890...21756651            ATTGACCAAATGGTG    AD001528 + 89 ESTs

TTCATACTTGGAATTGTATCCTTTGACCGTGACAT----TTCCTTGACTCCCTGTCCAGGTGGGTATTTTACAC
TTCATACTTGGAATT             21770488...21772086            GTGGGTATTTTACAC    AD001528 + 78 ESTs

TCGTCTGTGGCAACTGTATCCTCTCGGGAGCAGGG----CCTTAGCTAAACCCGCTCAGACATCCTGCTCAATG
TCGTCTGTGGCAACT            197775406...197776279           ACATCCTGCTCAATG    L33798   + 1  ESTs

CATTGTAGAATGGAAGTATCCTTCAGGGCCCGGGC----CTCCTTAACCCTGGCTCCAGGCCCTTCGAGACGAT
CATTGTAGAATGGAA            197811055...197812972           GCCCTTCGAGACGAT    L33798   + 1  ESTs

TTCCTGTAATCATGTGTATCCTTTTCAAAGGAAAT----TCCCTAACAAGATGCTTCAGCTTTCATGAATTCTG
TTCCTGTAATCATGT          chr4:154991334...154994285        CTTTCATGAATTCTG    RNF175 (AV725561 + 8  ESTs)

GATGATCAGTAATCCATATCCTTTAAAACAAAAAG----CCTCCTTAACTGAGATTTAGCTGGGAGCGCACACA
GATGATCAGTAATCC          chr4:154989247...154991231        CTGGGAGCGCACACA    RNF175 (AV725561 + 11 ESTs)

GGCACGAGCAGGAACGTATCCTTGAGACTGGTAGC----GTTCCTTAGCTATGGCGCAGAGCGGCGGCTGGAGC
GGCACGAGCAGGAAC          chr16:66473250...66484717         AGCGGCGGCTGGAGC    RCD-8 (L26339   + 28 ESTs)

TAATGGCAAAATTCAGTATCCATTCCTTCCTGTGG----GCCCTTAACACCCTGCTCAGAGAAGAGATCTTGGT
TAATGGCAAAATTCA          chr16:66468811...66480272         AGAAGAGATCTTGGT    RCD-8 (L26339   + 19 ESTs)
```

```
CATTCAACAAGCACCATATCCTTTTGGTTGTGAAA----GTCTTTAACTTATGATATACGACAGATGATTAATT
CATTCAACAAGCACC             36632115...36632941           GACAGATGATTAATT    D50926   + 18 ESTs

CGACCAAAATTTTTAGTATCCTTTTTCTGATTCCT----ATTTACCTTAATATTTTAAGTCTAAAACAGTGAGA
CGACCAAAATTTTTA             36638876...36639079           TCTAAAACAGTGAGA    D50926   + 4  ESTs

GCAACAGATAATCCTGTATCTTTTGATTGTATACT----GCCTTAACCTCTTTTGAAAGATGGTCTTTGCCAAT
GCAACAGATAATCCT             94877110...94880156           ATGGTCTTTGCCAAT    D16626   + 4  ESTs

AGAAGGAGTTTATCTGTATCCTTTCCCACAGGCTC----GTTCCTTTACCTGGATACAGATACAGCAAGTACCG
AGAAGGAGTTTATCT             94891072...94891178           ATACAGCAAGTACCG    D16626   + 2  ESTs

AGATCCTGGCTGTTGGTATCCTTAACGCCGCGTTG----TCCATTAACAATTCTCCCAGATATTAGCATTTGGT
AGATCCTGGCTGTTG            102296706...102302468          ATATTAGCATTTGGT    L20046   + 34 ESTs

CGACAAAATTAGAGAATATCCTTTGCTTCTTAAAA----TAAGTCTTAACTGCATGCATATTTTGTCAGCGGTA
CGACAAAATTAGAGA            102322750...102323609          ATTTTGTCAGCGGTA    L20046   + 34 ESTs

CCACCTCTGCTGTCTGTATCCTGCACAGCTGTGCT----TCCTTTATCCTCTGCTGCAGACATCAGCTCCGTTG
CCACCTCTGCTGTCT             44105778...44105921           ACATCAGCTCCGTTG    AB033002 + 4  ESTs

ATGGTGATGAAAACTGTATCCTGGAATTAAAATTG----TCTCCTTGACCGGCTCTCAGACATGGAGTTTCTCG
ATGGTGATGAAAACT             44119350...44119559           ACATGGAGTTTCTCG    AB033002 + 4  ESTs

GTGGTGACAGCTTTAATATCCTTTTGTTATATTTA----ACTAGCCTTAACAGTCTTACGTGACAAAGTTCACC
GTGGTGACAGCTTTA            134818263...134820627          GTGACAAAGTTCACC    AF030409 + 9  ESTs

GTAGTAGGAGCATTTGTATCCTTTATTATGTGTTT----TAACCTATTAATAATTTTAGGTTGCTATTTTCTTG
GTAGTAGGAGCATTT            134824438...134830264          GTTGCTATTTTCTTG    AF030409 + 8  ESTs

CAACTTTGATCATAAGTATCCTTAATTGAGGGAAA----CTCTTCCTTAACCACCGCAGCTATCTGAAGCCTCT
CAACTTTGATCATAA            134841173...134847754          CTATCTGAAGCCTCT    AF030409 + 10 ESTs
```
